# Incorporating parotid gland inhomogeneity into head‐and‐neck treatment optimization through the use of artificial base plans

**DOI:** 10.1002/acm2.13192

**Published:** 2021-02-09

**Authors:** Caleb M. Sample, Jonn Wu, Steven Thomas, Haley Clark

**Affiliations:** ^1^ Department of Medical Physics BC Cancer Agency Vancouver BC Canada; ^2^ Department of Physics and Astronomy University of British Columbia Vancouver BC Canada; ^3^ Department of Radiation Oncology BC Cancer Agency Vancouver BC Canada; ^4^ Faculty of Medicine University of British Columbia Vancouver BC Canada; ^5^ Department of Medical Physics BC Cancer Agency Surrey BC Canada

**Keywords:** base plan, constraint, head‐and‐neck, parotid, radiotherapy, xerostomia

## Abstract

Despite a great improvement in target volume dose conformality made possible in recent years by modulated therapies, xerostomia remains a common and severe side effect for head‐and‐neck radiotherapy patients. It is known that parotid glands exhibit a spatially varying dose response; however, the relative importance of subregions throughout the entire gland has yet to be incorporated into treatment plan optimization, with the current standard being to minimize the mean dose to whole parotid glands. The relative importance of regions within contralateral parotid glands has been recently quantified, creating an opportunity for the development of a method for including this data in plan optimization. We present a universal and straightforward approach for imposing varying sub‐parotid gland dose constraints during inverse treatment planning by using patient‐specific artificial base plans to penalize dose deposited in sensitive regions. In this work, the proposed method of optimization is demonstrated to reduce dose to regions of high relative importance throughout contralateral parotids and improve predictions for stimulated saliva output at 1‐year post‐radiotherapy. This method may also be applied to impose varying dose constraints to other organs‐at‐risk for which regional importance data exists.

## 1 INTRODUCTION

Intensity‐modulated radiotherapy (IMRT) has proven to be one of the most important advances in oncology in recent years,^1^ allowing a significantly higher precision of target volume dose conformality to be achieved in radiotherapy.^2^ However, parotid‐sparing IMRT alone is inadequate for complete sparing of salivary function,^3^ and it remains common for head‐and‐neck RT (radiotherapy) patients to be burdened by a severe loss in saliva production following treatment.^4^ Head‐and‐neck target volumes commonly exist adjacent to or overlapping with the parotid and submandibular glands,^5^ which along with sharp dose gradients and setup error, results in dose to salivary glands being probable during head‐and‐neck radiation treatment.^6^ Xerostomia (subjectively dry mouth) and hyposalivation (impaired salivary flow) significantly impact one's quality of life by crippling common abilities such as speech, chewing, swallowing, or tasting,^7^ while also causing oral infections, dental caries, and other oral sequela.^8^, ^9^, ^10^ Radiation to the parotid gland is the greatest risk factor for post‐treatment xerostomia.^11^

The ability of intensity‐modulated therapy treatment planning to attenuate the risk of xerostomia is dependent on what parotid gland dose constraints are passed to the optimizer. A constraint on the whole‐mean dose is effectual for preserving an OAR (organ‐at‐risk) which exhibits a pure parallel functional architecture, given that the spatial variance of dose within the OAR is unimportant. The parotid gland was once believed to exhibit a pure parallel architecture and hence a spatially homogenous dose response,^12^ and to this day, the current standard of care for minimizing the risk of post‐RT xerostomia incidence for head‐and‐neck patients is to constrain the whole‐gland mean dose.^13^ However, recent preclinical studies have demonstrated radiosensitivity within parotid glands to be inhomogeneous, having a spatially varying dose response throughout.^14^, ^15^, ^16^, ^17^, ^18^

In a recent study, a model including voxel dose data as well as patient demographic and clinical pathology features^14^ found the superior–anterior portion of the parotid gland to be the most influential in predicting xerostomia recovery. Furthermore, it was found that patients who developed xerostomia had a much higher mean dose to the inferior portion of the parotid gland.^14^ Another study^15^ used a tenfold cross validation test to show that dose to the region of the parotid gland containing stem/progenitor cells around the first branching of the Stensen's duct, was more predictive of xerostomia at 1 yr than dose to any other subregion of the gland. The same study also showed that the spatial distribution of dose in rat parotid glands affected salivary function recovery after treatment. Dose to the cranial 50% of the gland resulted in more than a 50% loss in salivary output, as well as tissue degeneration throughout the entire gland.

Clark et al.^16^ partitioned contralateral parotid glands (CPGs) for a single cohort of 332 patients into 2, 3, 4, 18, and 96 equal volume subsegments and derived the relative importance of each from mean dose regressors using random forests and conditional inference trees. The parotid gland with the lowest mean dose was defined as the CPG. Parotid gland structure sets and dose profiles were used to calculate the mean dose to various subsegments, and outcomes were described using stimulated saliva output at 1‐yr post‐RT and self‐assessed xerostomia questionnaires. For 18 subsegments, the most important subsegment (caudal–anterior) had a relative importance of 3.85 times the expected result for a homogenous parotid gland. The least important subregion exhibited virtually no importance.^16^

Clark et al.'s model was chosen to be used for implementing spatially varying dose constraints for multiple reasons. For one, it is the only available model that maps out relative importance values throughout the entire gland. Furthermore, it derives importance data within the original reference frame of the parotid gland without transforming to an alternate reference frame. Subsegmentation into 18 equal volume subsegments of CPGs creates a desirable size for varying constraints, as it is large enough for dose to be effectively steered, while small enough to account for the varying importance within the gland.

The spatial inhomogeneity of the dose response within the parotid gland, if incorporated into external beam RT treatment planning, could reduce the risk of xerostomia for head‐and‐neck patients. Studies have concluded that incorporation of nonhomogeneous effects into treatment planning can lead to improved outcomes.^19^, ^20^ The purpose of this work is to demonstrate the feasibility of a simple technique for including sub‐parotid gland importance data into RT treatment plan optimization using artificial base plans (BPs). To demonstrate the technique, we used Clark et al.'s^16^ intra‐parotid gland importance data.

## 2 MATERIALS AND METHOD

The RapidArc™ optimizer in Varian Eclipse™ (Varian Medical Systems, Inc.) is equipped with the ability to incorporate earlier radiotherapy deliveries into optimization. Pre‐existing spatial dose distributions can be loaded directly in as BPs during optimization, and the standard optimization workflow proceeds otherwise unaltered. We made use of this feature to apply a spatially varying dose constraint to the parotid gland to preferentially spare regions of high relative importance from excessive dose during radiotherapy.

**FIG. 1:**
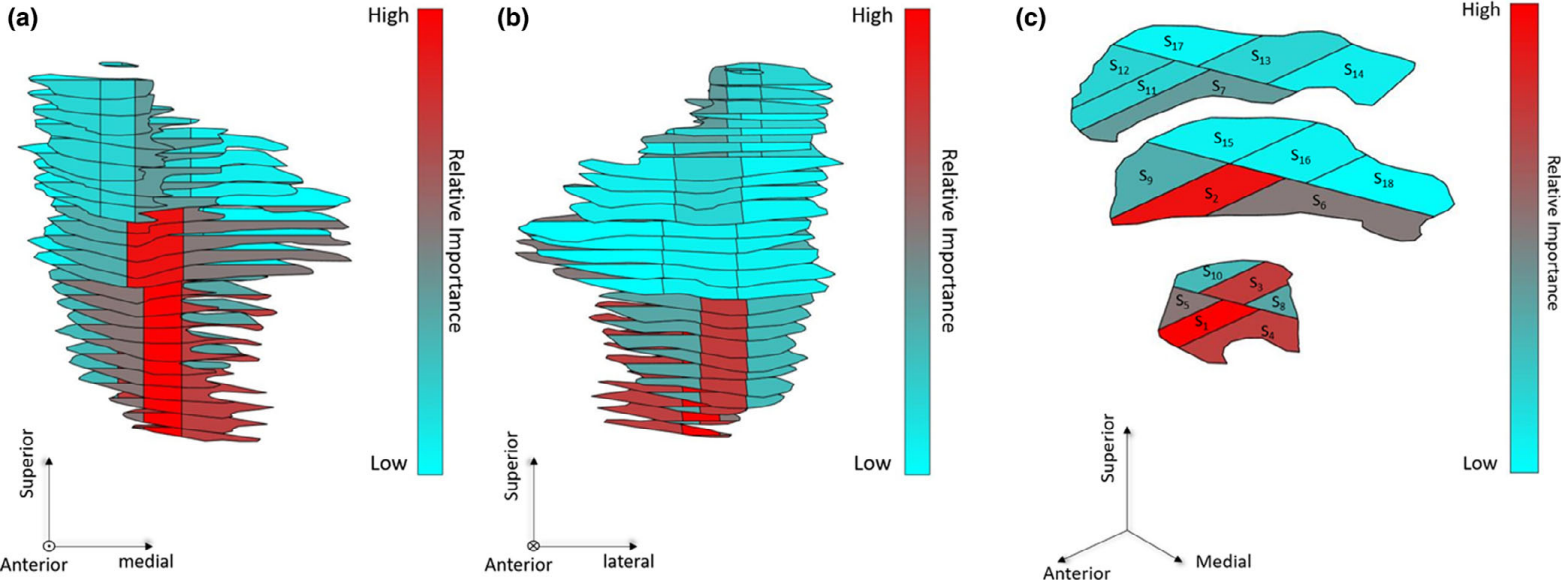
Subsegments of contralateral parotid glands have varying relative importance for predicting post‐RT xerostomia.^16^ Here, the spatial distribution of importance is illustrated from (a) the anterior, and (b) the posterior. Subsegments are labeled according to their importance in (c).

The radiotherapy structure set object of the DICOM standard files (DICOM‐RTSTRUCT) for 15 retrospective head‐and‐neck VMAT patients was exported from the ARIA^®^ Oncology Information database (primary tumor site: 5 tonsil, 4 tongue, 3 base of tongue, 1 nasopharynx, 1 thyroid, 1 left neck; prescription dose: 14 70 Gy/35 fractions, 1 60 Gy/25 fractions). Each patient received a single fractionation scheme using a simultaneous integrated boost. Sex and age statistics for the cohort were unknown as patients were previously anonymized. The median volume of primary PTVs was 181.5 cc (maximum: 295.1 cc, minimum: 30.18 cc). CPGs were defined as the parotid gland having the lowest mean dose in the original treatment plan for each patient and were subsegmented into 18 equal volume regions using DICOMautomaton.^21^ The average minimum distance between the primary PTV and the CPG was 3.3 cm. The relative importance of all 18 subsegments was determined using Clark et al.'s population‐level importance data.^16^ Subsegments were labeled in order of decreasing relative importance as $S_{1}\to S_{18}$, where $S_{1}$ is the subsegment of highest relative importance. A subsegmented CPG is shown labeled in Fig. 1.

DICOMautomaton was used to create artificial dose distributions (base plans) for each patient which adhered to the following formula:

1. Dose to all voxels located outside the CPG is zero.
2. Dose to regions of overlap between the CPG and target volumes is zero. This ensures that the prescription dose and tumor coverage will not be impacted.
3. Within each subsegment of the CPG, the dose is uniform
4. Dose to the region of highest relative importance (caudal‐anterior, Fig. 1 was $D_{0}$, and the dose to other subregions was $D_{0}\;I$, where I is a scaling factor proportional to the relative importance of the region compared to the most important subregion.

Five different types of BPs were created for each patient. $D_{0}$ was set to 10Gy, 20Gy, and 30Gy to create distributions with a linear scale I, and these BPs were named BP_10_, BP_20_, and BP_30_, respectively. The values of D_0_ were chosen as they span the range of typical mean parotid gland doses, and having multiple values allows us to empirically determine which type of base plan is most effective for applying constraints. In addition to the three BPs mentioned, a fourth was identical to BP_20_ for subsegments $S_{1}\to S_{5}$, while the dose to all other subsegments ($S_{6}\to S_{18}$) were zeroed; a fifth was assigned 50 Gy to subsegments $S_{1}\to S_{5}$, and 0 Gy to all other subsegments. These two plans were named BP_20,5_ and BP_top5_.

In Varian Eclipse™, each patient had five placeholder plans created for the five artificially constructed BPs that were imported for use in External Beam Planning. As a control, VMAT plans were retroactively optimized while adhering to standard clinical head‐and‐neck protocols (both parotids whole mean < 25 Gy or 1 parotid whole mean < 20 Gy). For each patient, two arcs with opposite direction 360° gantry rotations and a difference of 60° in collimator rotation (30° and 330°) were used. Plans were then reoptimized using each artificial BP. Loading the BPs into the optimizer does not by itself implement a spatially varying dose constraint throughout the CPG, as the standard parotid dose constraint is on the whole‐mean dose. Therefore, an additional upper bound dose constraint must be placed on the CPG. This constraint, combined with the BP dose, provides a spatially varying dose constraint which preferentially restricts dose to subsegments of high relative importance. The ideal constraint to set depends on the individual anatomy of the patient and was chosen to be between 0 and 15 Gy over the maximum dose in the current BP. In this manner, the constraint imposed on a given region of the CPG has varying strength, depending on the region's dose in the BP. All clinical dose constraints were met for all plans. These plans are named P_10_, P_20_, P_30_, P_20,5_, and P_top5_, corresponding to the use of BP_10_, BP_20_, BP_30_, BP_20,5_, and BP_top5_. The control plan optimized without a BP is referred to as P_0_.

To maximize validity of a comparison between different plan types, it was paramount to minimize interplan bias and dose variability within structures other than the CPG. All plans for a given patient were optimized toward approximately the same V98 (percent volume receiving at least 98% of the prescription dose) in the closest PTV to the CPG. Dose constraints to other OARS for any given patient were optimized according to clinical guidelines and were independent of plan type, and PTV coverage was adjusted minimally. Doses to all OARs without PTV overlap were kept below the standard clinical constraints. Each plan for a given patient had the same constraint on the whole‐mean dose of the CPG, and plans using BPs which had an upper bound constraint all had the same D_max_–D_0_. For example, if P_10_ had the constraint D_max_ < 20 Gy, then P_20_ had the constraint D_max_ < 30 Gy. These constraints remained constant throughout the course of optimization.

A C# console application for calculating mean dose to subsegments and exporting data was developed and tested to create a plug‐in script that can run through the Eclipse™ Scripting API in Varian Eclipse™. A MATLAB script was then created to analyze parotid gland subsegment and other structure doses for the 15·6=90 plans.

**FIG. 2:**
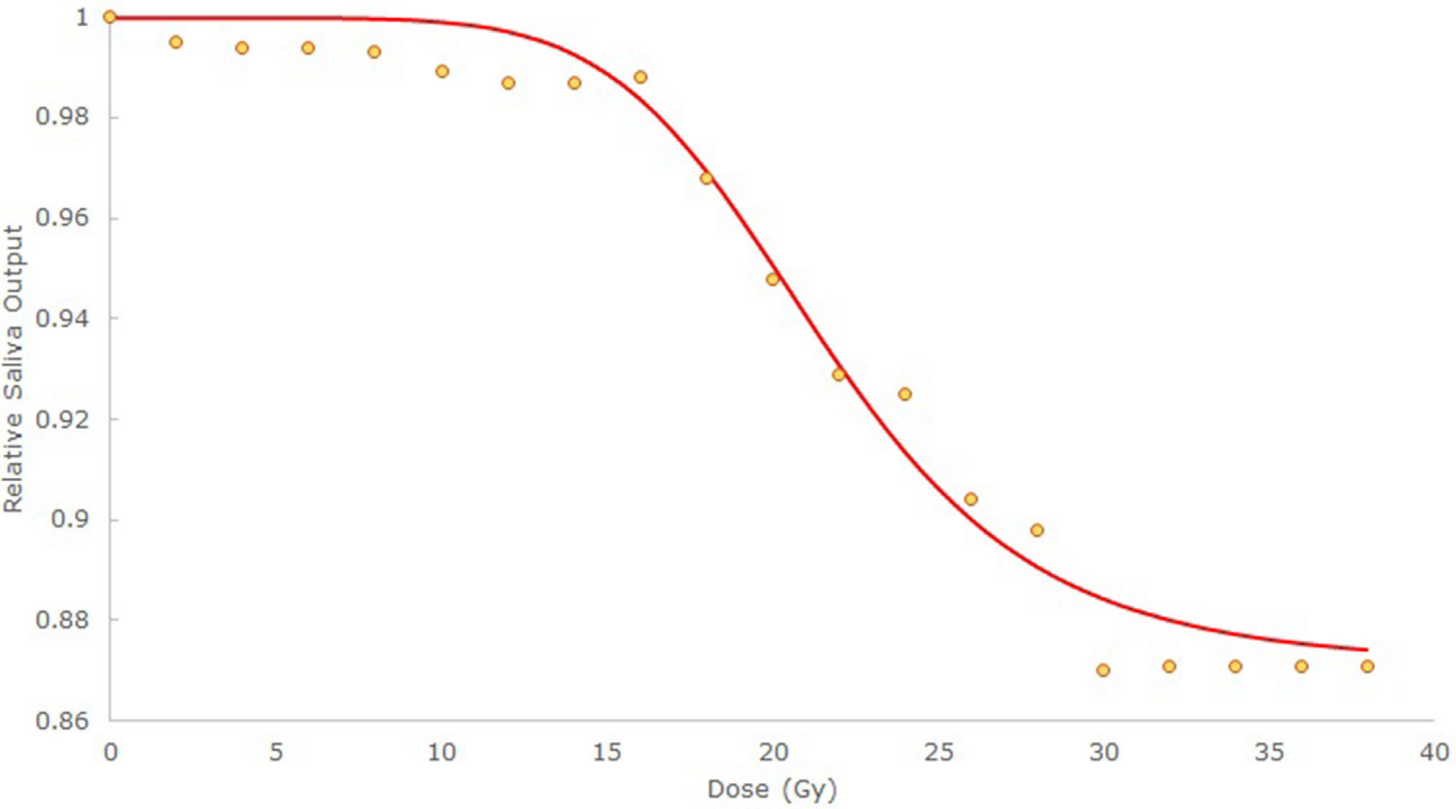
The predicted stimulated saliva output according to the Clark et al.^16^ model at 1‐yr post‐RT relative to baseline is shown for subsegment $S_{1}$.

Mean subsegment dose reductions between plans optimized with BPs were quantified and compared to determine which BP was most effective, and if significant reductions in mean dose to critical regions occurred. Dose to all patient structures for each plan was evaluated to ensure that all clinical dose constraints were met, and to determine if the use of BPs significantly altered mean or maximum doses to other structures. Significance was assessed at P=0.05 with a paired t test. Mean subsegment doses for each plan were passed as parameters into a predictive model for stimulated salivary output at 1yr relative to baseline adapted from Clark et al.^16^ To reduce noise in the model, a dose response curve (Hill Model^22^) was generated for each individual subsegment using the method of least squares. Curves were fit to data points obtained by incrementing dose to each subsegment from 0 by 2 Gy to 40 Gy while keeping all other subsegment doses constant. A final predictive model of the form

(1) $$S\left(D_{1},\;D_{2},\;\ldots \;D_{17},\;D_{18}\right)=1‐\sum_{i=1}^{18}\Delta_{i}\left[1‐\frac{1}{1+{\left(\frac{D_{i}}{D_{50{,}_{i}}}\right)}^{n_{i}}}\right]=1‐\sum_{i=1}^{18}\Delta_{i}\frac{1}{1+{\left(\frac{D_{50,i}}{D_{i}}\right)}^{n_{i}}}$$

was created, where $D_{i}$ is the dose to subsegment $S_{i}$, $\Delta_{i}$ is the maximum loss in salivary output predicted by infinite dose to $S_{1}$ independently, and $D_{50_{i}}$, $n_{i}$ are parameters fit to the data, representing the dose predicting a decline in salivary output of $\frac{1}{2}\Delta_{i}$, and the steepness of the curve. The predicted response when only dose to $S_{1}$ is considered (Eq. (1) with only i=1) is shown in Fig. 2.

## 3 RESULTS

**FIG. 3:**
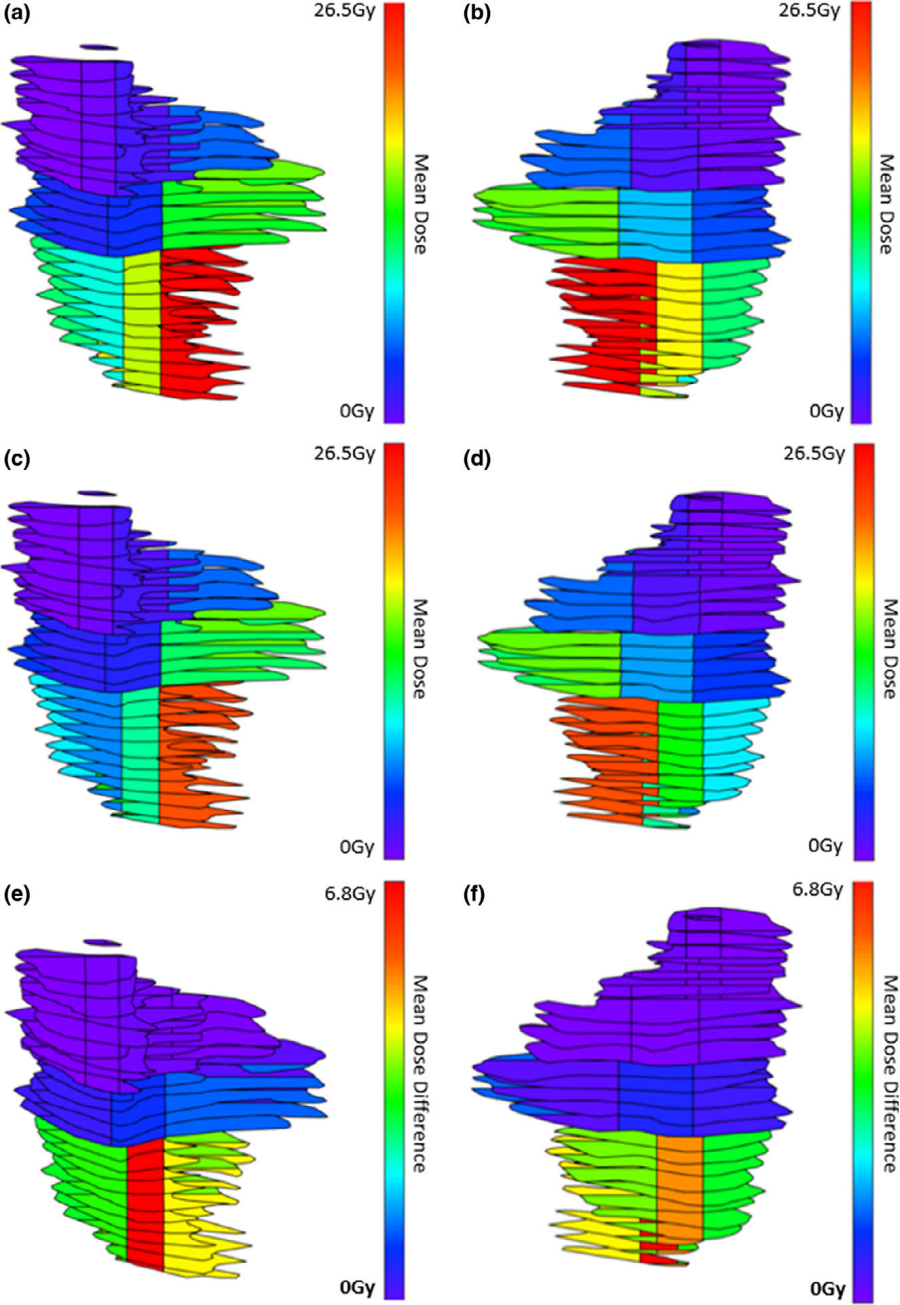
Statistically significant reductions in dose to the top five most important subsegments of the contralateral parotid gland ($S_{1}$ → $S_{5}$) were obtained using BP_20_, BP_30_, and BP_top5_ during optimization. (a) and (b): mean subsegment doses for plans optimized without BPs (anterior and posterior view); (c) and (d): mean subsegment doses for plans optimized with BP_30_ (anterior and posterior view); (e) and (f): mean difference in dose between plans optimized with and without BPs (anterior and posterior).

Within the CPG, the mean dose to all subsegments is shown for each plan type in Table 1 with statistically significant dose differences indicated. P_top5_ showed the greatest reduction to the mean dose of $S_{1}$ (M = 8.6 Gy, SD = 3.9 Gy), t(14) = 8.62, *P* = <0.001, followed closely by P_30_ (M = 8.1 Gy, SD = 3.7 Gy), t(14) = 8.45, *P* < 0.001. All plans reduced the whole mean dose of the CPG significantly, with the greatest reduction seen with P_30_ (M = 2.1 Gy, SD = 1.2 Gy), t(14) = 6.60, *P* < 0.001), followed by P_20_ (M = 2.0 Gy, SD = 1.0 Gy), t(14) = 7.68, *P* < 0.001). In general, dose to parotid gland subsegments of high relative importance, which tends toward the caudal end of the gland, was reduced when planning with BPs as seen in Fig. 3. P_20_, P_30_, and P_top5_ significantly reduced dose to the top five most important subsegments of the CPG, while P_10_ and P_20,5_ insignificantly reduced dose to S_2_. The mean dose received by each subsegment of the CPG in each plan type is listed in Table 1. P_top5_ significantly increased dose to several subsegments of low relative importance (S_13_, S_14_, S_16_, S_17_, S_18_).

**TABLE 1:** The mean dose in each subsegment of the contralateral parotid gland for each plan type is shown. A subscript “s” represents a significant (*P* < 0.05) reduction in dose, while subscript “si” represents a significant increase in dose.

Subsegment	Mean dose (Gy)					
	Plan type					
	P_0_	P_10_	P_20_	P_30_	P_20,5_	P_top5_
S_1_	27.8	22.5**^s^**	20.9**^s^**	19.8**^s^**	20.7**^s^**	19.2**^s^**
S_2_	11.1	10.8	9.9**^s^**	10.0**^s^**	10.9	10.0**^s^**
S_3_	29.6	24.6**^s^**	23.8**^s^**	23.3**^s^**	23.4**^s^**	21.5**^s^**
S_4_	40.2	36.2**^s^**	35.5**^s^**	34.7**^s^**	35.2**^s^**	33.2**^s^**
S_5_	18.3	15.7**^s^**	14.6**^s^**	14.2**^s^**	14.8**^s^**	13.5**^s^**
S_6_	22.3	21.5**^s^**	20.8**^s^**	20.9^s^	22.6	22.8
S_7_	8.3	8.3	8.2	8.3	9.0	9.4
S_8_	40.8	37.2**^s^**	36.8**^s^**	36.3**^s^**	36.9**^s^**	35.5**^s^**
S_9_	10.2	10.0	9.5	9.7	10.4	10.1
S_10_	20.9	17.7**^s^**	17.2**^s^**	17.1**^s^**	17.4**^s^**	15.6**^s^**
S_11_	6.5	6.5	6.4	6.6	7.0	7.1
S_12_	5.9	5.97	5.8	6.0	6.4	6.6
S_13_	7.8	7.7	7.8	7.9	8.45	9.0**^si^**
S_14_	12.6	12.3	12.8	12.7	13.5	14.6^si^
S_15_	12.4	12.1	11.7	11.8	12.6	13.3
S_16_	15.7	15.0**^s^**	14.7**^s^**	14.9	15.8	17.2**^si^**
S_17_	6.8	6.8	6.9	6.8	7.4	7.9**^si^**
S_18_	25.3	24.5**^s^**	25.0	25.1	26.0	27.8**^si^**
Whole gland	18.3	16.7**^s^**	16.3**^s^**	16.2^s^	16.8^s^	16.6^s^

**FIG. 4:**
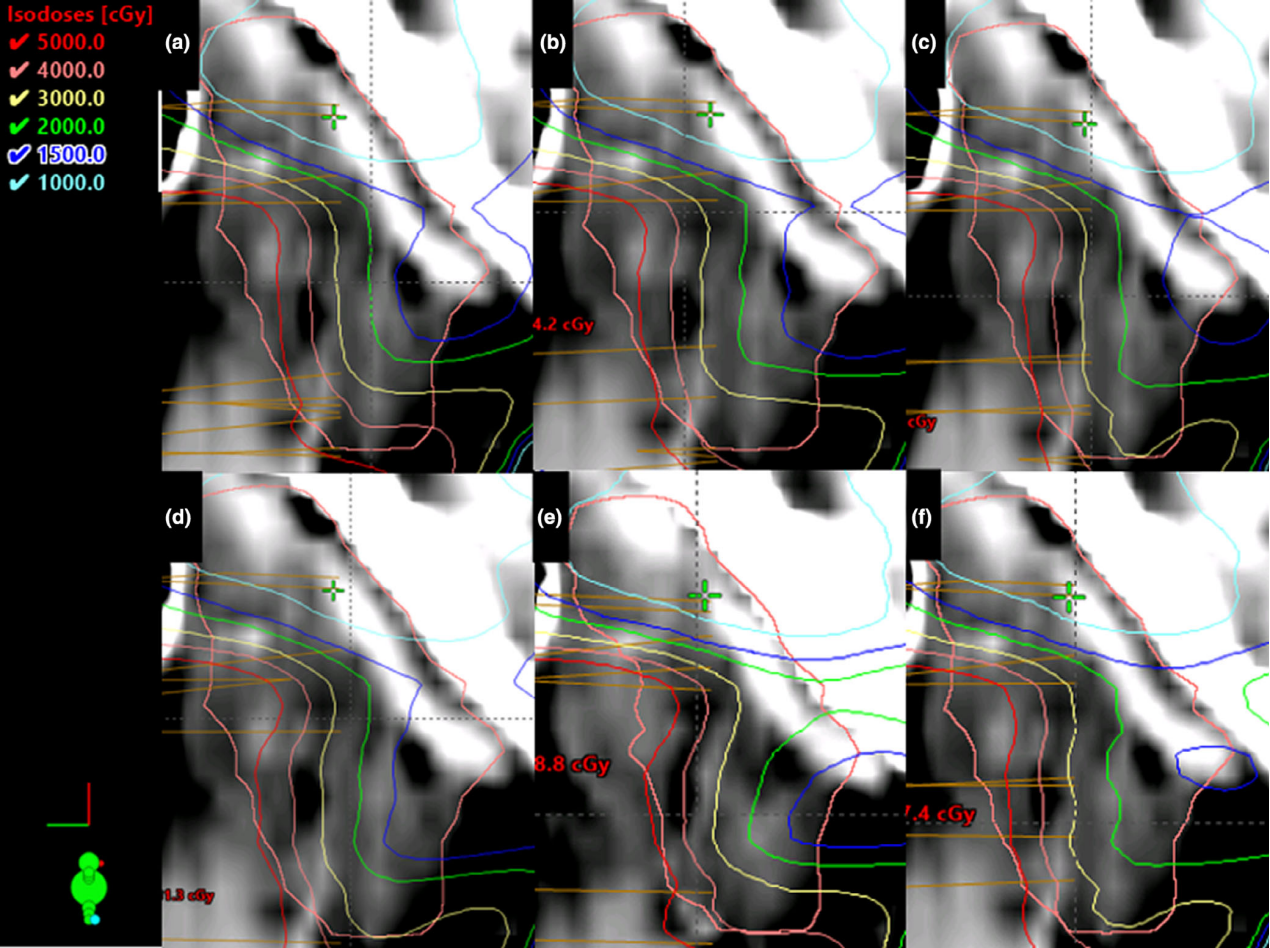
The dose distribution for a sagittal plane slice of the CPG is shown for each different plan type created for a single patient. (a): P_0_; (b): P_10_; (c): P_20_; (d): P_30_; (e): P_top5_; (f): P_20,5._ The most important subsegment derived with Clark et al.'s model is located in the caudal–anterior (bottom right) portion of the gland, where isodose lines can be clearly seen to shift away from when using BPs, and particularly with BP_30_ (D).

All plans optimized with BPs demonstrated statistically significant improvements in stimulated saliva predictions at 1‐year post‐RT. Optimizing with BPs resulted in up to a 23% improvement in predicted saliva output (mean = 18%) as compared to optimizing without BPs, as shown in Table 2. BP_30_ and BP_Top5_ demonstrated the greatest improvements in salivary output, while BP_10_ resulted in the smallest improvement (13%). One patient's dose distribution in the middle of the CPG is shown for each type of plan in Fig. 4.

**TABLE 2:** Stimulated saliva output predictions from a population based model^16^ are shown for each plan type.

Plan type	Saliva output (fraction of baseline)	Improvement from P_0_ (%)	Absolute saliva output increase (%)	Statistical significance
P_0_	0.48	N/A	N/A	N/A
P_10_	0.54	13	6	t(14) = 3.1, *P* < 0.01
P_20_	0.57	19	9	t(14) = 5.3, *P* < 0.001
P_30_	0.59	23	11	t(14) = 4.7, *P* < 0.001
P_20,5_	0.55	15	7	t(14) = 2.9, *P* < 0.02
P_top5_	0.59	23	11	t(14) = 4.0, *P* < 0.002

The mean overlap percentage of CPGs with target volumes was 13.7% (median 13%, maximum 33%), and subsegments along the caudal–medial portion of glands were most prone to overlap. The frequency of overlapping for various subsegments is summarized in Table 3.

**TABLE 3:** The number of patients with contralateral parotid gland subsegments overlapping with planning target volumes.

Subsegment	Number Overlapping	Subsegment	Number
$S_{1}$	11	$S_{10}$	4
$S_{2}$	1	$S_{11}$	0
$S_{3}$	11	$S_{12}$	0
$S_{4}$	13	$S_{13}$	0
$S_{5}$	2	$S_{14}$	4
$S_{6}$	11	$S_{15}$	0
$S_{7}$	4	$S_{16}$	8
$S_{8}$	13	$S_{17}$	0
$S_{9}$	0	$S_{18}$	11
Whole gland	13		

**FIG. 5:**
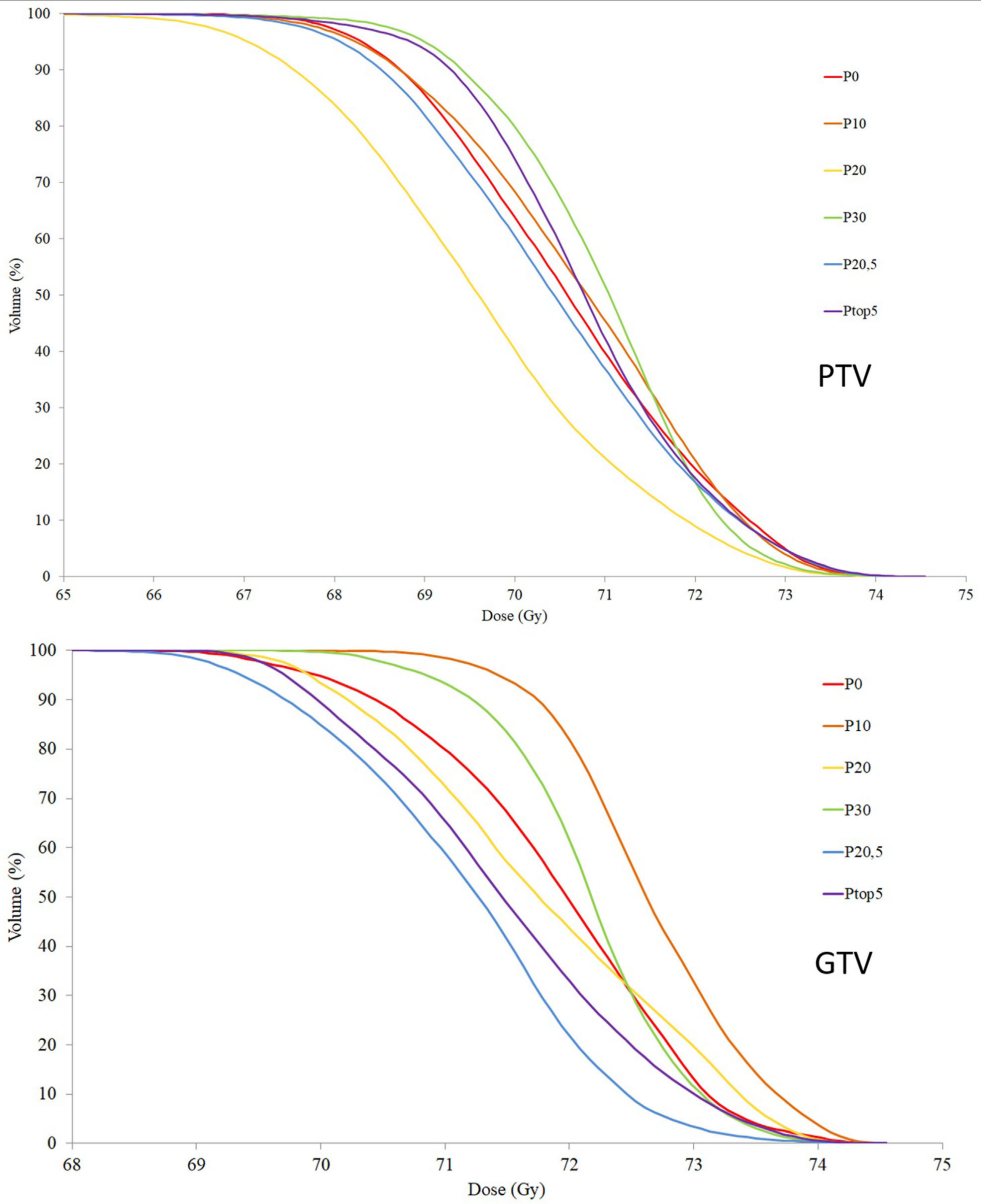
The cumulative dose–volume histogram for the primary PTV (a) and gross tumor volume (GTV, b) revealed no apparent trend toward decreased or increased coverage when BPs are used during optimization, as illustrated with a single representative patient.

Optimizing with BPs did not prevent clinical dose constraints for OARs or target volumes from being adhered to. Primary and secondary PTVs had no apparent trend toward decreased or increased dose coverage with base plans. The cumulative DVH for one patient's primary PTV is shown for all different plan types in Fig. 5. Submandibular glands had extensive overlap with target volumes in all 15 patients, making it impossible for them to be spared from high dose without subsequent reductions in dose to target volumes. Contralateral submandibular gland mean doses were increased with statistical significance when optimizing with BPs, with P_Top5_ resulting in the largest difference (M = 1.7 Gy, SD = 1.5 Gy), t(10) = 3.86, *P* < 0.01. Three patients’ contralateral submandibular glands were not contoured and were not included in this statistic. Mean dose to the oral cavity was also increased with statistical significance when using BPs, with the largest difference found with P_20_ (M = 1.4 Gy, SD = 1.7 G).

## 4 DISCUSSION

The base plan approach for incorporating various intra‐parotid gland dose constraints into head‐and‐neck RT plans through the use of artificially constructed dose distributions demonstrated that the optimizer can effectively steer dose away from critical regions. This method does not require creation of additional structure contours, or manipulation of unique dose constraints for the numerous subsegments, allowing the optimization workflow to proceed normally after loading the base plan and setting the one additional constraint on the CPG. Furthermore, adding a unique dose constraint for each subsegments structure may have a noticeable impact on the optimization time.

We used population‐level relative importance information of 18 equal volume parotid gland subsegments,^16^ derived from a large cohort of patients to decide upon various suborgan dose constraints to impose during optimization. This approach produced more favorable predicted patient outcomes. Predicted xerostomia risk was lowered with minimal additional effort required during treatment planning. This method improves upon the current whole‐parotid approach to treatment planning by incorporating parotid dose response heterogeneity into treatment plan optimization.

That parotid gland subsegments of high relative importance showed significant reductions in dose, while less important subsegments had more modest improvements or even increases in dose, is not unexpected. Highly important subsegments had proportionally high dose constraints set so that dose could be redirected away from important regions without implementing too strict a constraint on the whole gland such that PTV coverage is compromised. Regions of high relative importance tend toward the caudal end of the CPG, where the largest reductions in mean dose were found. The extent of PTV overlap with CPGs has been shown to be correlated with patient outcomes^23^, ^24^, ^25^ and the base plan approach has less impact when patients have extensive overlap. CPGs were frequently found to overlap with planning target volumes in this study, with 13/15 parotid glands involving some degree of overlap. Despite this difficulty, the whole‐mean dose and mean dose to important subsegments frequently found to overlap with target volumes (S_1_, S_3,_ S_4,_ S_6_) showed significant reductions in dose with P_30_.

Five different types of base plans were used to reoptimize treatment plans in this study. The two most effective plans for sparing important subsegments and improving predicted patient outcomes were P_30_ and P_top5_, which resulted in identical predictions for stimulated saliva output at 1‐year post‐RT. The fact that these were the most effective plans is unsurprising as they offer the largest variance in dose constraints within the CPGs. P_top5_ marginally outperformed P_30_ in terms of reducing dose to the top five most important subsegments; however, this came at the expense of statistically significant increases in dose to other parotid subsegments of low relative importance. While this is presumably a favorable trade‐off, given uncertainty in the model P_30_ is a more moderate plan which also achieved the greatest reduction to the whole mean dose of the CPG, qualifying it as the plan adhering best to the ALARA (As Low As Reasonably Achievable) principle.

P_30_ is also favorable over P_top5_ as it is easier to manage within Varian Eclipse™. The base plan approach presents challenges when imposing large dose penalties, as the maximum dose contained in the base plan will contribute to the maximum body dose during optimization, which could impede the planner's ability to determine if a dose constraint for the body is violated or not. Another subtlety which must be accounted for during optimization is a shift in the CPG mean dose constraint that occurs when using BPs. The CPG's mean dose in the BP is absorbed into the mean dose of the gland during optimization, so the planner must shift the whole‐mean dose of the CPG up by an amount equal to the mean dose within the BP. Both of these issues can be mitigated by copying the optimized fields to a new plan without BPs, but it might not immediately be clear to reviewers whether a BP has been used or is included in dose reports.

A significant reduction in dose to the top five important regions of CPGs as well as the whole mean as seen with P_30_ demonstrates efficacy of the proposed technique. Dose to other OARs and target volumes continued to meet clinical constraints when planning with BPs. Increases in dose to contralateral submandibular glands were not clinically significant, albeit did have statistical significance. The dose to these glands was either weakly constrained or not at all, and as a result, small increases in their already high doses had no effect on patient outcomes. Oral cavity mean doses were kept below clinical recommendations, so the statistically significant increase in mean dose seen is likely to be clinically insignificant.

The model by Clark et al.^16^ has yet to be clinically validated since it was derived. We believe it to be a valid quantitative model as saliva predictions using segmentation into 18 subregions had values for mean‐absolute‐error and root‐mean‐square‐error which are comparable to values when predictions were made using whole‐mean dose.^16^ Clark et al.'s model was used in our methods as it was the most favorable for designing constraints,however, the base plan method for imposing dose constraints can be applied using data from other models of regional importance for an organ at risk. The emphasis of this work is on the method of incorporating subregional dose constraints using artificial base plans that are specifically designed based on model data.

A challenge in this study was establishing a valid interplan comparison, as the varying initial dose conditions contained in each base plan ensures that optimized dose profiles for plans are nonidentical both inside and outside the CPGs. To minimize systematic errors, dose constraints to all OARs other than the CPG and all PTVs other than those in proximity to the CPG were set to the same value in different plan types, and the same V98 goal for bordering/overlapping PTVs was set in each case. Plans were created in a random order by a single planner for all patients. However, variability in the optimization process for each plan was impossible to eliminate entirely and could have impacted this study.

In the future, we wish to extend this work by imposing suborgan optimization criteria directly through the scripting API of treatment planning systems. The prospect of including dose constraints without requiring artificial base plans is appealing, but presents additional challenges as Varian Eclipse™'s scripting API requires that automatic optimization proceeds inside a separate scripting window, meaning that dose constraints must be rigidly preset by the treatment planner. On the contrary, the approach presented here allows the treatment planner full and regular access to the standard optimization window, so that constraints can be adjusted throughout the course of optimization.

## 5 CONCLUSION

It is known that parotid glands exhibit an inhomogeneous dose response, and there is promise that post‐treatment xerostomia outcomes can be significantly improved by incorporating this information into clinical planning workflows. A universal method for incorporating suborgan dose constraints into VMAT treatment planning has been featured as an effective means of steering dose away from important regions of the parotid gland. This method may also be applied to other OARs for which spatial importance data exist.

## AUTHOR CONTRIBUTION STATEMENT

All authors were involved in designing this study. The manuscript was drafted by Caleb Sample, and critical revisions were made by Haley Clark, Jonn Wu, and Steven Thomas. Software for developing base plans and predicting saliva output was generated by Haley Clark. Treatment planning and analysis was done by Caleb Sample.

## CONFLICT OF INTEREST

Caleb Sample, Jonn Wu, Steven Thomas, and Haley Clark have no conflict of interest to disclose.
